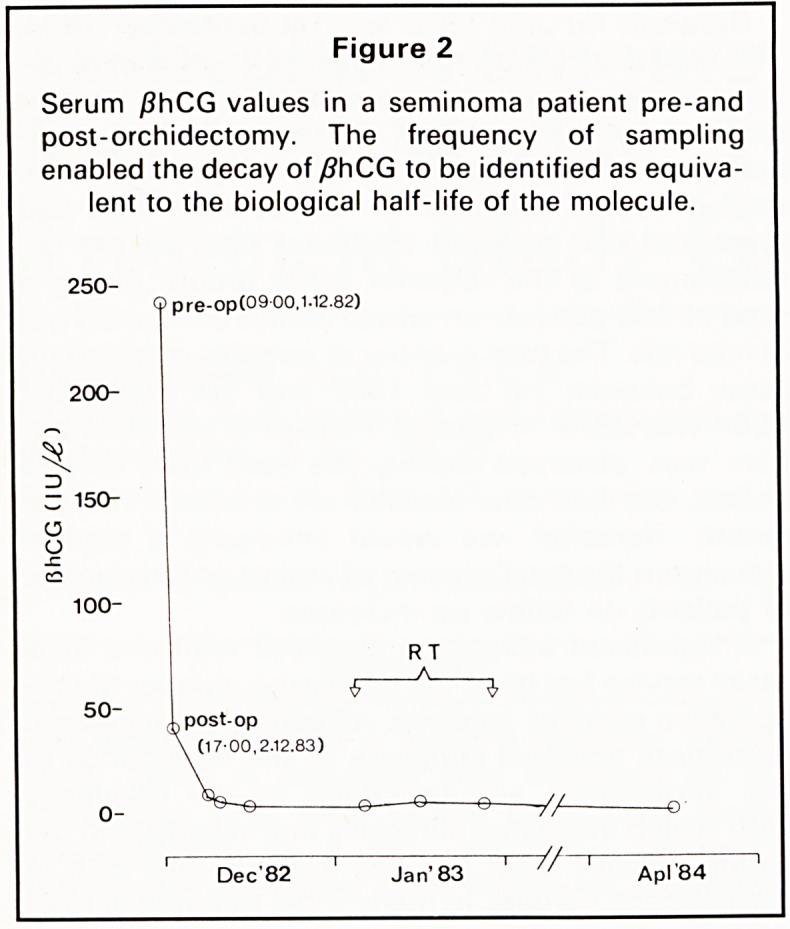# Testicular Tumours, Treatment Study Group

**Published:** 1985-01

**Authors:** Harold Eckert, P. Ann Light

**Affiliations:** Bristol Radiotherapy and Oncology Centre, Bristol Royal Infirmary; Bristol Radiotherapy and Oncology Centre, Bristol Royal Infirmary

**Keywords:** Testicular cancer, Tumour markers, Alpha-foetoprotein, Human chorionic gonadotrophin

## Abstract

The establishment of a regional testicular tumour study group is described with emphasis on the importance of a local facility for tumour marker measurements by a fully integrated laboratory. Advantages over larger scale studies are suggested.


### Testicular Tumours: Establishment of a Regional Marker Assay Service and a Treatment Study Group

**Harold Eckert, M.D., F.R.C.R.**
Consultant Radiotherapist and Oncologist
**P. Ann Light, B.Sc., Ph.D.**
Senior Biochemist, Bristol Radiotherapy and Oncology Centre, Bristol Royal Infirmary

*Keywords*: Testicular cancer; Tumour markers; Alpha-foetoprotein; Human chorionic gonadotrophin

### SUMMARY

The establishment of a regional testicular tumour study group is described with emphasis on the importance of a local facility for tumour marker measurements by a fully integrated laboratory. Advantages over larger scale studies are suggested.

### INTRODUCTION

The incidence of testicular cancer has been rising steadily since the early years of this century, and it is now the commonest form of cancer in men aged 25–34 (Davies, 1981). In the United Kingdom it affects approximately one man in 500 before the age of 50. However, during the past few years the prognosis for these patients has improved substantially chiefly as a result of the introduction of effective chemotherapy especially the drug cisplatin, and also because of improved clinical staging procedures, and the development of rapid, sensitive radioimmunoassay techniques for the measurement of the tumour markers α-foetoprotein (AFP) and human chorionic gonadotrophin (hCG).

**Figure 1**

Increased incidence of testicular cancer observed at the Bristol Radiotherapy Centre.

### WHY A 'REGIONAL STUDY GROUP'?

The South West Region of England comprises a population of approximately 3 million, and the most recent detailed statistics available for this area (1975–77) (S.W.R.H.A. Med. Stats. Bureau, 1981) indicate an annual incidence of testicular cancer similar to the overall national figure, i.e. 3.3 per 100,000 males. However, our own more recent data indicate that the incidence is now significantly greater than this (Figure 1) and continues to increase, having almost doubled in incidence since that time.

In view of the comparative rarity of these tumours and the need for early assessment of new forms of treatment, it is clear that collaborative studies between groups of treatment centres afford the most practical way of monitoring results. The South West Region contains four centres for radiotherapy and oncology; however, its size and shape mean that each centre serves a catchment area and a population comparable with many other provincial regional centres in the U.K. Additionally, the small number of junior staff in training (there are only two senior registrars in the entire region) mean that these patients are closely supervised by consultant staff who are members of the study group, providing continuity of care and experience. Since it is not feasible to attempt to treat all S.W. testicular cancer patients in just one regional specialist centre, it was decided in 1981 that the four centres should enter into a collaborative study of the management of testicular teratocarcinomas. In such a relatively small group of patients, controlled clinical trials are not possible. A careful study of a standardised treatment protocol with frequent monitoring of results of treatment and of the problems associated with it therefore affords the best opportunity to advance our knowledge of the management of this disease. This was, in part, made possible by the existence in Bristol of an M.R.C. funded clinical trials unit able to assist in data collection through a computerised records system.

### MEMBERSHIP OF THE STUDY GROUP

The group membership comprises a radiotherapist from each collaborating centre, a urological surgeon and a medical oncologist, as well as a pathologist, a diagnostic radiologist and the biochemist responsible for marker measurements. The administrative facility is provided by the clinician and the secretary to the M.R.C. trials unit.

### PROTOCOL FOR THE STUDY

The first task of the group was the agreement of a standardised protocol for the investigation and treatment of these patients with the object of improving pre-treatment assessment and staging, establishing a standardised approach to treatment and assessing response, relapse and survival, as well as the morbidity of treatment.

A simple registration form was devised for use by the surgeons. Once this has been received by the trials unit subsequent details are obtained from radiotherapy departments and the histological sections are reviewed.

### WHY A REGIONAL MARKER ASSAY SERVICE?

An integral requirement for such a regional testicular tumour study group was believed to be the provision of a regional marker assay service. This was established in the Bristol Radiotherapy and Oncology Centre and its role and advantages have since been vindicated.

The study group and the marker assay service were both formally established in July 1981, and during the initial period radiotherapists, medical oncologists, urological surgeons and clinical biochemistry laboratories throughout the South West Region were notified of its existence and invited to collaborate in the study by sending patient details to the M.R.C. office for registration and serum samples for marker assay to Bristol instead of to the Supraregional Assay Service in London. By mid 1982, a pattern of serum sample numbers and frequency had emerged, and a clearly defined workload established.

Between 1st July 1982 and 1st September 1984, 169 new testicular tumour patients have been registered in the study group and their serum samples received for marker assay. Of these, 76 had seminomas and 93 had teratomas. Marker assays are also carried out on testicular tumour patients who had presented and received treatment prior to the establishment of the regional study group, giving a total of 356 patients on whom assays are carried out at intervals. The total number of samples received for assay between 1st July 1982 and 1st September 1984 was 2874. A gradual increase in sample numbers was observed during the early part of this period, but has now levelled off at about 120 per month. Hereafter, we would anticipate a gradual increase in the total number of assays as the number of patients on follow up increases.

A significant advantage observed with this local assay service has been the increasing number of preoperative samples received, reflecting the increased awareness amongst surgeons of the importance of this information, and facilitated by the frequency with which interested clinicians and scientists in the region actually meet together and are thus able to discuss such matters. In many other reported studies on testicular tumour markers, the value of the data has been severely impaired by the absence of preoperative levels (Read et al, 1983).

Preoperative marker measurements are of great value in the subsequent management of patients. In patients from whom no preoperative data have been obtained and who have subsequently been found to have normal marker values, it is not possible to know if marker levels had been elevated before removal of the tumour, and hence whether subsequent tumour regrowth might be indicated by raised values.

Prior to the establishment of this local assay service, all marker assays were carried out by the Supraregional Assay Service at Charing Cross Hospital, London. This inevitably resulted in a very much greater time interval between the sample date and the report date. A clear advantage of a local assay service is the speed with which results can be obtained and the information transmitted to the clinician. Although such very rapid results are not routinely required, it is important to be able to select potentially urgent candidates and process their samples swiftly. An example of a situation in which speed of assay proved valuable is that of a patient diagnosed following orchidectomy as a seminoma of the testis. Marker assays were carried out preoperatively and a raised hCG detected (Figure 2). This information was telephoned at once to the surgeon, with a request that subsequent blood samples be taken frequently. This frequent sampling enabled it to be demonstrated that the serum hCG concentration fell to normal with the theoretical biological half-life of the molecule (approximately

**Figure 2**

Serum $\beta$hCG values in a seminoma patient pre-and post-orchidectomy. The frequency of sampling enabled the decay of $\beta$hCG to be identified as equivalent to the biological half-life of the molecule.

36 hours), providing strong evidence for the complete removal of tumour by surgery. This was subsequently supported by radiological investigations.

The acquisition of rapid results has proved of great value in a number of analogous cases, and also in patients who have had a disease-free interval following their initial therapy, but who then present with ill-defined symptoms of a possible recurrence.

A very important advantage observed in our local assay service has been that of the collection of serial data on individual patients and its correlation with their clinical progress. This has proved particularly useful in the recognition of 'unexpected' results – for example, when an apparently well patient on routine follow up suddenly produces a slightly elevated marker value, this information can be telephoned immediately to the clinician and further measurements, investigations and therapy be instigated with the minimum of delay.

Another important aspect which has emerged from acquisition of such serial data combined with clinical information is the recognition of previously unnoticed or unreported aspects of the disease. One such on-going study concerns anomalous serum hCG elevations which appear not to correlate with disease (Light et al., 1982). Another study underway is an investigation of the ratio of intact molecular hCG to the free $\beta$ sub-unit in serum; markedly differing ratios have been found in some patients, and have already been published (Light et al., 1983).

### CONCLUSIONS

A regional organisation for the study and treatment of rare tumours such as testicular terato-carcinoma affords an excellent opportunity to provide data on treatment effectiveness or morbidity without the impersonal and often less well adhered to regimens which are the frequent result of very large multicentre studies.

We have found the size of our organisation to be large enough to collect a reasonable number of patients and to provide our own marker estimation facility economically. It is, however, small enough to permit regular meetings between all the participants which also allows detailed discussion of the proposed methods of treatment providing a conformity which may not be so readily obtained in larger studies. Additionally, some groups of patients provide a ready-made pool for collaboration with larger national studies, such as the Medical Research Council Surveillance Study in early cases.

In this group of patients the most valuable outcome has been the provision of the means to estimate tumour markers frequently and to obtain results rapidly during the course of treatment. Furthermore, anomalies can be more readily observed and interpreted through the close liaison between laboratory and clinician, and the personal interest which this engenders in the progress of each patient.

The value of this study will increase as the information obtained is collated and analysed, and we will be able to compare our own data with that of other workers in this field.

### ACKNOWLEDGEMENTS

We are grateful to the following members of the Study Group for their invaluable collaboration: Professor E. Rhys Davies, Dr. J. D. Davies, Dr. E. D. Gilby, Dr. S. E. Goodman, Dr. D. J. Mahy, Dr. C. G. Rowland, Mr. P. J. B. Smith and Dr. C. J. Tyrrell. We also wish to thank the Medical Research Council for their part in establishing the Trials Unit in Bristol, the Cancer Research Campaign for establishing a similar unit in Plymouth, and Bristol-Myers for financial support.

### REFERENCES

1. DAVIES, J. M. (1981) Testicular cancer in England and Wales: some epidemiological aspects. *Lancet* **i**, 928–932.
2. South Western Regional Health Authority Medical Statistics Bureau (1981) Cancer in the South West; Statistics for 1975–77.

3. READ, G., JOHNSON, R. J. WILKINSON, P. M., EDDLESTON, B. (1983) Prospective study of follow up alone in stage 1 teratoma of the testis. *Br.Med.J.* **287**, 1503–1505.
4. LIGHT, P. A., FELTON, T. and ECKERT, H. (1982) False-positive markers in testicular tumours. *Lancet* **ii**, 1214.
5. LIGHT, P. A., FOSTER, J. P., FELTON, T., ECKERT, H. and TOVEY, K. C. (1983) Molecular heterogeneity of chorionic gonadotrophin in some testicular cancer patients. *Lancet* **i**, 1284.

## Figures and Tables

**Figure 1 f1:**
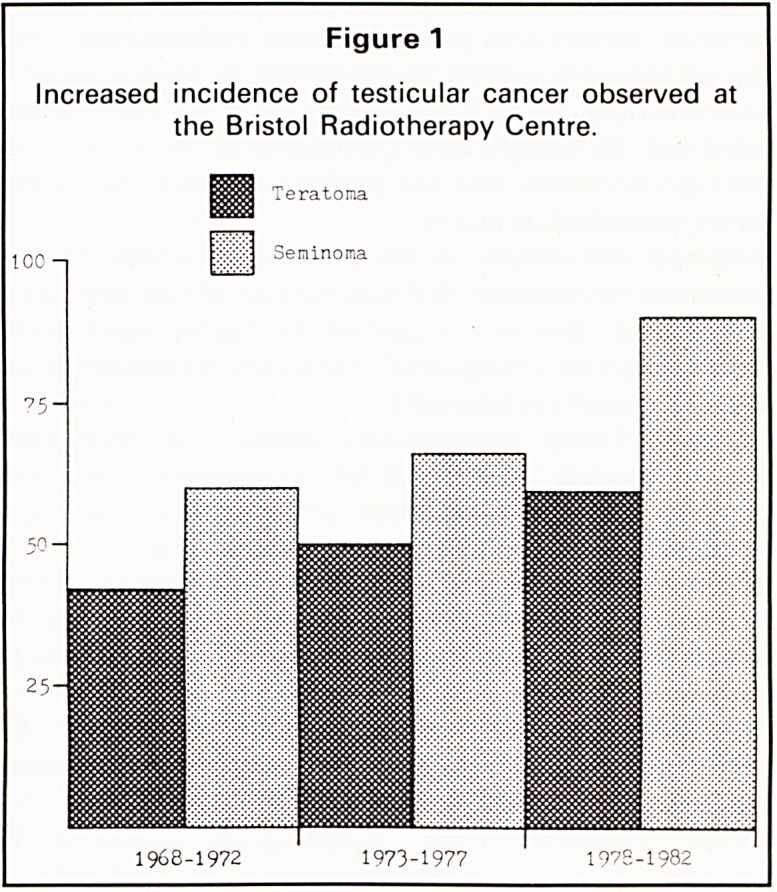


**Figure 2 f2:**